# Supplementary material for: Mesenchymal MACF1 Facilitates SMAD7 Nuclear Translocation to Drive Bone Formation
Source: Cells. 2020 Mar 4;9(3):616. doi: 10.3390/cells9030616 (PMC7140458; doi:10.3390/cells9030616)
Supplement: Supplementary file 1 [file cells-09-00616-s001.pdf]

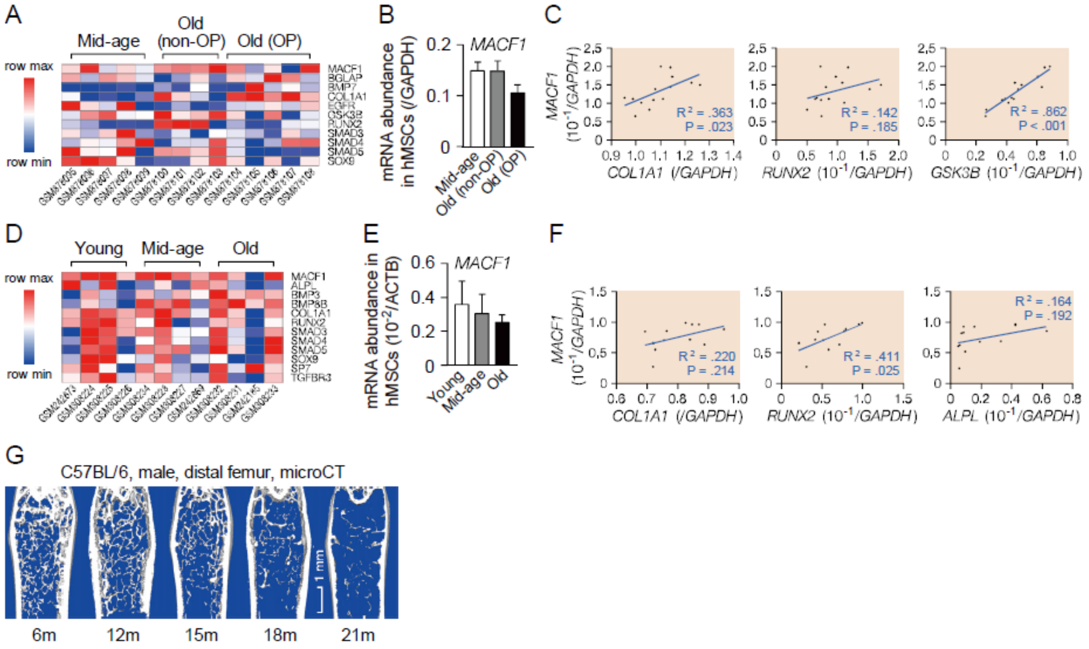

Figure S1. Mesenchymal MACF1 expression is correlated with attenuation of osteogenic differentiation induced by primary osteoporosis. (A) Gene expression profile of MSCs from healthy, elderly, and elderly osteoporotic patients (GSE35959). (B) *MACF1* relative abundance in MSCs of healthy and osteoporotic patients. (C) Correlation analysis of MACF1 and osteogenic genes. (D) Gene expression profile of MSCs from young, middle-aged, and elderly patients (GSE12274). (E) *MACF1* relative abundance in MSCs of young, middle aged, and elderly patients. (F) Correlation analysis of *MACF1* and osteogenic genes. (G) Representative microCT 3D reconstructed images showing distal femur microarchitecture in wildtype mice at different ages.

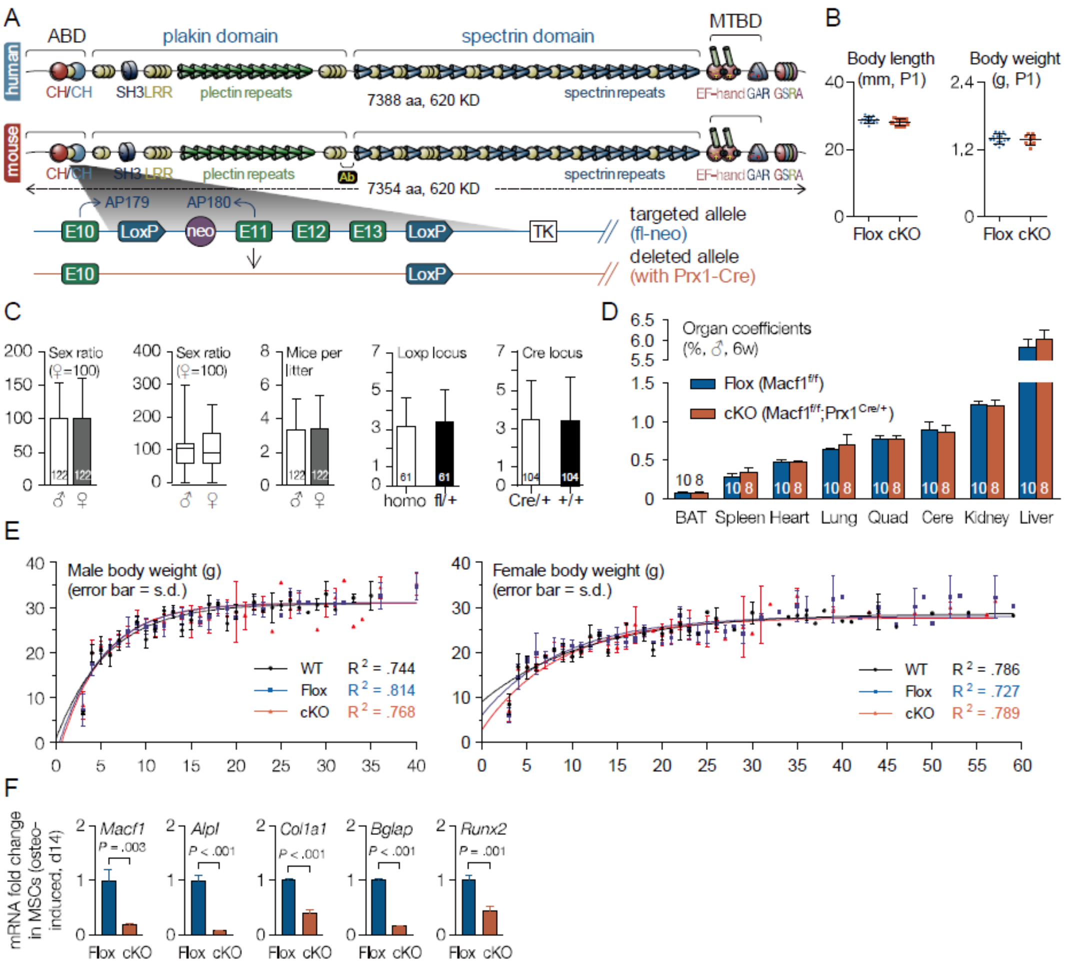

Figure S2. Mesenchymal deletion of MACF1. (A) Schematic diagram showing domain structure of the human and mouse MACF1 protein (UniProtKB: Q9UPN3-1, Q9QXZ0-1). Targeting strategy for generating the MACF1 conditional knockout (cKO) mice is also provided. CH—calponin homology; SH3—the SRC homology 3 domain; LRR—leucine-rich repeat; GAR—gas2 (growth arrest specific 2)-related domain; GSRA—tandem repeats of Gly/Ser/Arg/Ala; ABD—actin binding domain; MTBD—microtubule binding domain; Ab—recognition site of the anti-MACF1 antibody (abcam, ab117418); E10—exon 10; Neo—neomycin resistance cassette; TK—thymidine kinase cassette; AP179/180—arbitrary primers designed for genotyping; Prx1-Cre—paired related homeobox 1 promoter driven Cre recombinase expressing mouse. (B) Body length and weight of the neonatal MACF1 cKO mice (P1, postnatal day 1). Body length was defined as ventral distance between the mouth and anus. (C) Ratio of segregation of genes in the offspring. Data were collected within 122 litters. (D) Organ coefficients of 6-week old male mice. Data were normalized by body weight. BAT—brown adipose tissue; Quad—quadriceps. Cere—cerebrum. (E) Growth curves of male and female MACF1 cKO mice. Least-squares fitting Gompertz function was used. (F) Real-time PCR analysis of osteogenic marker genes in osteo-induced cKO MSCs (d14). Data are represented as mean± s.d. Gene expression was normalized by *GAPDH*/*Gapdh*. Significances were determined using Student's *t*-test.

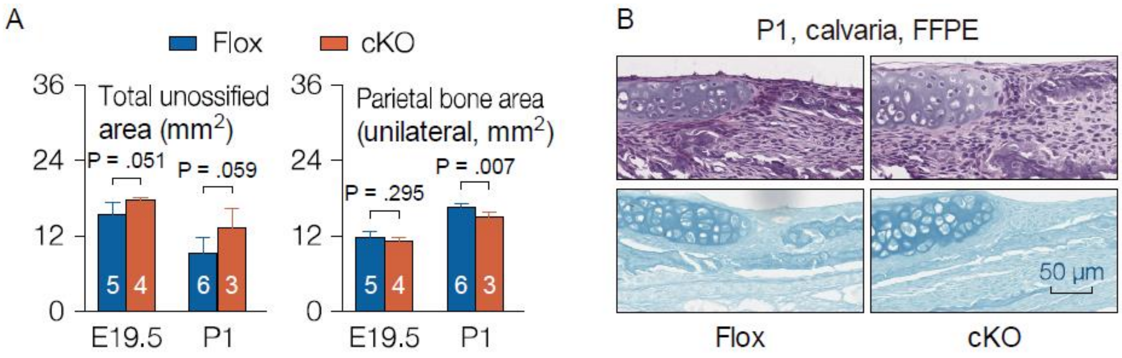

Figure S3. Mesenchymal deletion of MACF1 retards early stage bone development in mice. (A) Additional quantification for skeletal preparation of the skull in Figure 2 panel A. (B) Representative images of Alcian blue staining showing morphology of chondrocytes in calvaria at P1. Data are represented as mean ± s.d., and are compared with littermate controls. Statistical significances were determined using Student's *t*-test.

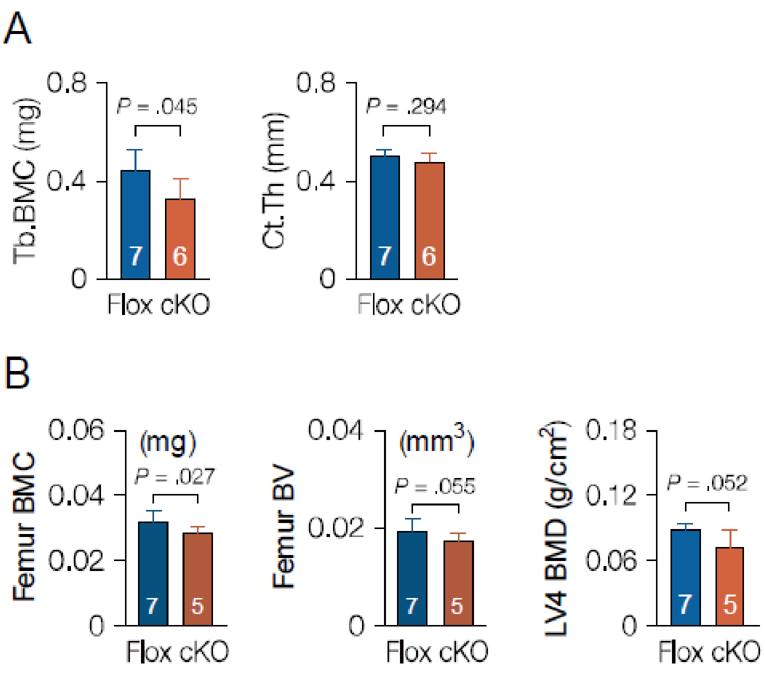

Figure S4. Mesenchymal deletion of MACF1 weakens bone properties in adult mice. (A) Additional quantification of microCT analysis in Figure 3 panel A. (B) Additional quantification of DEXA scanning in Figure 5 panel A. Data are represented as mean ± s.d. Statistical significance were determined using Student's *t*-test.

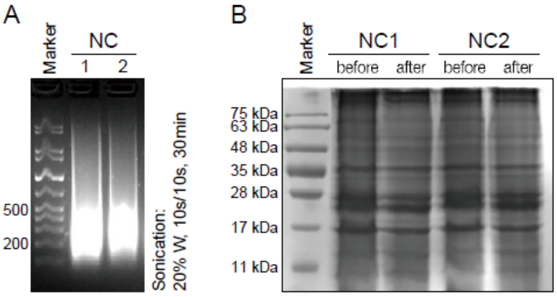

C

Input gene	Gene ID	Input gene	Gene ID	Input gene	Gene ID
Fn1	2335	Pdgfa	5154	Vcam1	7412
Gdf10	2662	Phex	5251	Vdr	7421
Gdf11	10220	Runx2	860	Vegfa	7422
GDF2	2658	Serpinh1	871	Vegfb	7423
GDF5	8200	Smad1	4086	Wnt1	7471
GDF6	392255	Smad2	4087	Wnt10A	80326
GDF7	151449	Smad3	4088	Wnt10B	7480
Gli1	2735	Smad4	4089	Wnt11	7481
GSK3A	2931	Smad5	4090	Wnt16	51384
GSK3B	2932	Smad6	4091	Wnt2	7472
Icam1	3383	Smad7	4092	Wnt2B	7482
Igf1	3479	Smad9	4093	Wnt3	7473
Igf1r	3480	SMURF1	57154	Wnt3A	89780
IGF2	3481	SMURF2	64750	Wnt4	54361
Ihh	3549	Sost	50964	Wnt5A	7474
ITGA1	3672	Sox9	6662	Wnt5B	81029
Itga2	3673	Sp7	121340	Wnt6	7475
Itga2b	3674	Spp1	6696	Wnt7A	7476
Itga3	3675	Tgfb1	7040	Wnt7B	7477
Itgam	3684	Tgfb2	7042	Wnt8A	7478
Itgav	3685	Tgfb3	7043	Wnt8B	7479
Itgb1	3688	Tgfbr1	7046	Wnt9A	7483
Mmp10	4319	Tgfbr2	7048	Wnt9B	7484
Mmp2	4313	Tgfbr3	7049		
Mmp8	4317	Tnf	7124		
Mmp9	4318	TNFRSF11B	4982		
Nfkb1	4790	Tnfsf11	8600		
Nog	9241	Twist1	7291		

Input gene	Gene ID	Input gene	Gene ID	Input gene	Gene ID	Input gene	Gene ID
Acvr1	90	Bmp5	653	Chrd	8646	Csf2	1437
Ahsg	197	Bmp6	654	Col10a1	1300	Csf3	1440
Alpl	249	Bmp7	655	Col14a1	7373	CTNNB1	1499
Anxa5	308	BMP8a	353500	COL15A1	1306	Ctsk	1513
Bglap	632	BMP8b	656	Col1a1	1277	Dlx5	1749
Bgn	633	Bmpr1a	657	Col1a2	1278	Egf	1950
Bmp1	649	Bmpr1b	658	Col2a1	1280	EGFR	1956
BMP10	27302	Bmpr2	659	Col3a1	1281	Fgf1	2246
BMP15	9210	CALCR	799	Col4a1	1282	Fgf2	2247
Bmp2	650	Cd36	948	Col5a1	1289	Fgfr1	2260
Bmp3	651	Cdh11	1009	Comp	1311	Fgfr2	2263
Bmp4	652	Cebpa	1050	Csf1	1435	Flt1	2321

D

Rank	Gene Symbol	Gene ID	GO: Molecular Function		GO: Biological Process		GO: Cellular Component		Human Phenotype		Mouse Phenotype		Pathway		Pubmed		Disease		Average Score	Overall pValue	Interactant count	Score
			Score	P value	Score	P value	Score	P value	Score	P value	Score	P value	Score	P value	Score	P value	Score	P value				
1	SMAD7	4092	1	.00039	1	.09005	.99018	.01665	.96196	.03815	1	.04822	1	.00407	1	.03970	1	.03195	.99402	.00007	104	.00066
2	REL	5966	.99753	.01123	1	.09005	.78245	.08443	N/A	0	1	.04822	.88625	.02033	1	.03970	1	.03486	.96037	.00179	224	.00024
3	HES1	3280	.99616	.01278	1	.09005	.53917	.13749	N/A	0	1	.04822	.67410	.03311	1	.03970	1	.03195	.90833	.00268	52	.00009
4	BHLHE40	8553	.99966	.00445	1	.09682	.73668	.09624	N/A	0	1	.04822	.30427	.06158	1	.03970	.97084	.07591	.89620	.00285	28	.00006
5	ZFP748	212276	DATA NOT FOUND																			

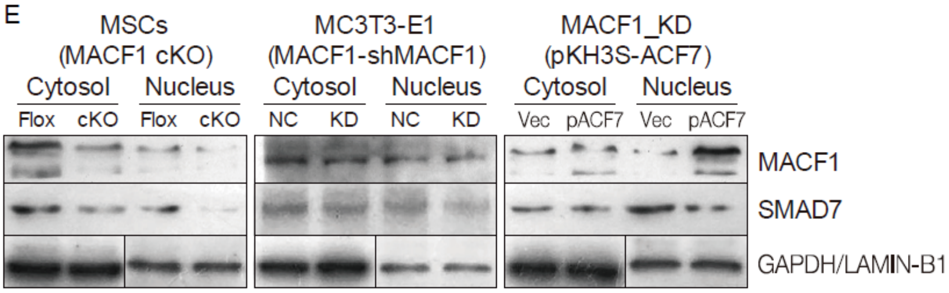

Figure S5. MACF1 interacts with SMAD7. (A) Gel electrophoresis for analysis of DNA quality in MC3T3-E1 lysate after sonication. (B) Coomassie blue staining for determination of protein quality in MC3T3-E1 lysate before and after sonication. (C) The 127 osteogenic-related genes used as the training set in the ToppGene database. (D) Candidate gene prioritization based on functional annotations and protein interactions using the ToppGene database. The *Score* value represents the similarity of a queried gene with genes in the training set. (E) Western blot analysis of MACF1 and SMAD7 levels in MACF1 cKO MSCs, MACF1 knockdown (KD) preosteoblasts, and MACF1 stable-overexpressed KD cells. ACF7 is a synonym for MACF1. Lamin B1 and GAPDH were used as internal reference for nucleus and cytoplasm, respectively.

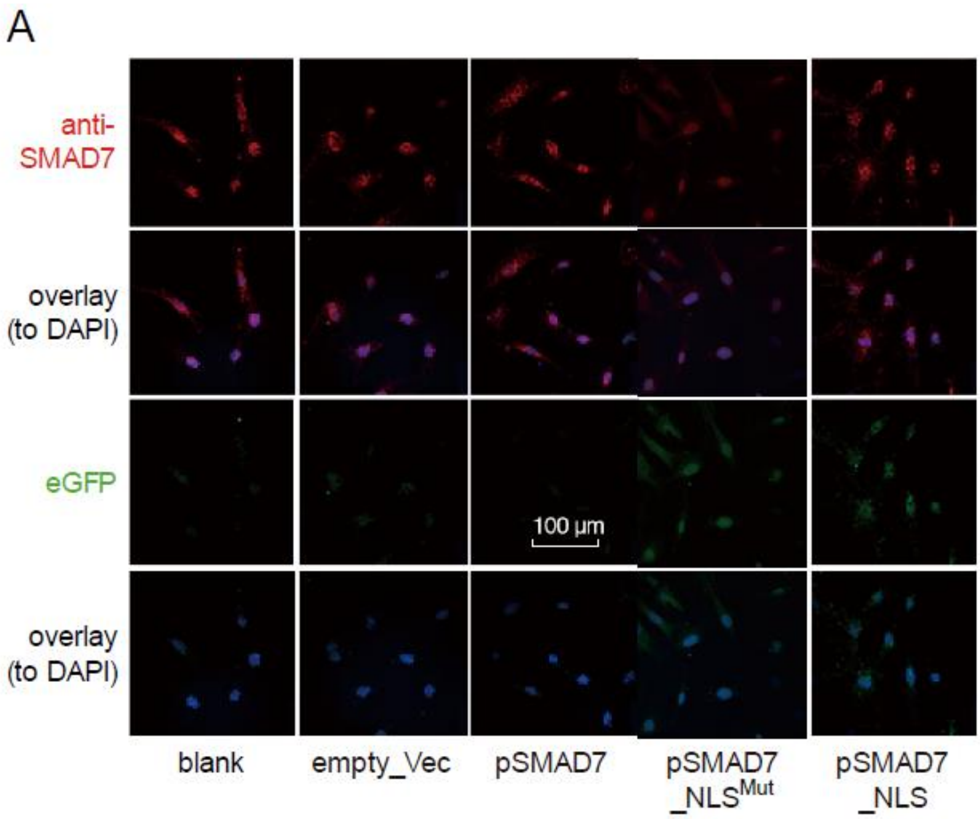

Figure S6. MACF1 facilitates SMAD7 nucleus translocation to drive osteogenic differentiation. (A) Representative immunostaining images of MC3T3-E1 preosteoblasts showing SMAD7 (red) and eGFP (green) expression 12h after plasmid transfection. The blank group was not transfected.
